# ATRX has a critical and conserved role in mammalian sexual differentiation

**DOI:** 10.1186/1471-213X-11-39

**Published:** 2011-06-14

**Authors:** Kim Huyhn, Marilyn B Renfree, Jennifer A Graves, Andrew J Pask

**Affiliations:** 1 ARC Centre of Excellence for Kangaroo Genomics, Australia; 2 Department of Zoology, The University of Melbourne, Victoria, 3010, Australia; 3 Research School of Biological Sciences, The Australian National University, ACT, 2601, Australia; 4 Department of Molecular and Cellular Biology, The University of Connecticut, Storrs, CT 06269 USA

**Keywords:** Marsupial, eutherian, tammar wallaby, testis, ovary, germ cells

## Abstract

**Background:**

X-linked alpha thalassemia, mental retardation syndrome in humans is a rare recessive disorder caused by mutations in the *ATRX *gene. The disease is characterised by severe mental retardation, mild alpha-thalassemia, microcephaly, short stature, facial, skeletal, genital and gonadal abnormalities.

**Results:**

We examined the expression of *ATRX *and *ATRY *during early development and gonadogenesis in two distantly related mammals: the tammar wallaby (a marsupial) and the mouse (a eutherian). This is the first examination of *ATRX *and *ATRY *in the developing mammalian gonad and fetus. *ATRX *and *ATRY *were strongly expressed in the developing male and female gonad respectively, of both species. In testes, *ATRY *expression was detected in the Sertoli cells, germ cells and some interstitial cells. In the developing ovaries, *ATRX *was initially restricted to the germ cells, but was present in the granulosa cells of mature ovaries from the primary follicle stage onwards and in the corpus luteum. *ATRX *mRNA expression was also examined outside the gonad in both mouse and tammar wallaby whole embryos. *ATRX *was detected in the developing limbs, craniofacial elements, neural tissues, tail and phallus. These sites correspond with developmental deficiencies displayed by ATR-X patients.

**Conclusions:**

There is a complex expression pattern throughout development in both mammals, consistent with many of the observed ATR-X syndrome phenotypes in humans. The distribution of ATRX mRNA and protein in the gonads was highly conserved between the tammar and the mouse. The expression profile within the germ cells and somatic cells strikingly overlaps with that of DMRT1, suggesting a possible link between these two genes in gonadal development. Taken together, these data suggest that ATRX has a critical and conserved role in normal development of the testis and ovary in both the somatic and germ cells, and that its broad roles in early mammalian development and gonadal function have remained unchanged for over 148 million years of mammalian evolution.

## Background

The *ATRX *gene is located on the mammalian X-chromosome and is a member of the SNF-2-like helicase superfamily subgroup that contains genes involved in DNA recombination, repair and regulation of transcription [1]. Mutations in this gene cause ATR-X syndrome in humans, a sex-linked condition characterized by alpha thalassaemia, severe psychomotor retardation, characteristic facial features, microcephaly, short stature, cardiac, skeletal and urogenital abnormalities [2,3]. Urogenital abnormalities occur in 80% of patients and range from complete male to female sex reversal [4], commonly associated with truncations of the C-terminus of the protein, to mild hypospadias [3]. However, the precise role of ATRX in gonadal development in mammals remains unclear. Although XY ATR-X patients have varying degrees of gonadal dysgenesis, a common feature is an absence of Müllerian ducts and differing degrees of virilization [5] showing that there is initial testis formation that subsequently becomes dysgenetic. The initial development of the testes indicates that the phenotype is not caused by sex reversal, but rather by early testicular failure and a subsequent lack of androgen. The absence of Müllerian ducts in affected individuals confirms the initial development of testes with functional Sertoli cells that are able to produce AMH (required for regression of the Müllerian ducts). Thus ATRX acts downstream of the sex-determining gene, *SRY *and of *SOX9 *that is required for Sertoli cell development and upregulation of *AMH *in testicular development [6]. This is consistent with the analysis of testes from ATR-X patients with more mild gonadal phenotypes. Such testes typically show reduced numbers of seminiferous tubules and functional Leydig cells but an absence of germ cells [4,7-9]. Together these phenotypes suggest that there is an early failure to maintain a viable testis causing reduced virilization. Depending on how early in development testicular failure occurs, the developmental effects can range from severe to mild feminization [10].

The mouse X-linked *ATRX *gene shares 85% homology with its human orthologue [11]. However, marsupials are unique in that they have orthologues of *ATRX *on both the X (*ATRX*) and Y (*ATRY*) chromosomes. *ATRX *shares 72% and 78% sequence identity with mouse and human respectively [12], while ATRY shares 61% sequence identity and 88% amino acid similarity with human and mouse ATRX [13]. The more divergent *ATRY *gene is functionally specialized, and is exclusively expressed in the male urogenital system, whereas *ATRX *has a broad pattern of mRNA expression in marsupials, as in mice and humans [6]. The only site where *ATRX *and *ATRY *are co-expressed in the marsupial is in the adult testis [6]. *ATRX *is present in the somatic and germ cells of the adult testes of humans and rats, indicating a possible role in spermatogenesis [14].

Orthologues of *ATRX *have also been identified in the nematode *Caenorhabditis elegans *(*C.elegans*), suggesting it is an ancient and ultra-conserved gene in the sex determination cascade. The *C.elegans ATRX *orthologue (*xnp-1*) has a remarkable conservation of function in gonadogenesis with human ATRX. *xnp1 *and *lin35 *(the orthologue to mammalian retinoblastoma) act in concert to regulate target gene expression in *C.elegans*. Their roles appear to be redundant since knockout of either *xnp1 *or *lin35 *alone does not produce a phenotype. However, the double mutant has abnormalities, which, despite their divergence from humans, paralleled those seen in XY ATR-X patients. The mutants were sterile with severe defects in gonadal development and a decrease in germ cell numbers [15]. Many of the male *C.elegans *also exhibited defects in the structure and/or function of the male mating apparatus [15]. These abnormalities are analogous to the gonadal dysgenesis and external genitalia defects of XY ATR-X patients [5]. The mutant *C.elegans *also had other consistent characteristics with human ATR-X patients including patterning abnormalities and growth restriction [5,15,16]. There were also gonadal anomalies in female *C.elegans *double mutants, suggesting a potential role in ovarian development.

To date there are very few reports detailing ATRX expression in mammals, and none that examine its role in the early gonad. Marsupials provide a unique system to examine the function of this gene particularly in reference to gonadal development since there are two orthologues, *ATRX *on the X-chromosome, and ATRY on the Y-chromosome. To further define the role of this gene in early gonadal development in mammals we examined expression of both ATRX and ATRY in the developing marsupial gonad and of ATRX in the developing mouse gonad.

## Results

The ATRX antibody used in this study (rabbit anti-human, Santa Cruz biotechnology, ATRX-H-300) binds with high affinity to both ATRX and ATRY in marsupials and as such cannot differentiate between the two orthologues. However, since only ATRY (and not ATRX) is expressed in the developing testes of marsupials [6], *in situ *and immunohistochemistry data in developing tammar wallaby testes demonstrates ATRY mRNA/protein distribution. Conversely, since ATRY is absent in XX females, all data in developing tammar ovaries represents ATRX mRNA/protein distribution. Similarly in mice, in which there is no Y-linked ATRY orthologue, all expression data represents ATRX mRNA/protein distribution. The only tissue in which ATRX/ATRY expression cannot be distinguished is in the adult tammar testis where the two genes are co-expressed [6]. A summary of *ATRX *and *ATRY *expression in the gonads of eutherian and marsupial mammals is shown in Table 1.

**Table 1:** Summary of *ATRX *and *ATRY *expression in the gonads of eutherian and marsupial mammals

Tissue	Atrx Mouse	ATRX Tammar	ATRY Tammar
Bi potential XY gonad	Yes Germ and somatic cells	No	Yes Germ and somatic cells

Developing Testis	Yes Sertoli, germ and Leydig cells	No	Yes Sertoli, germ and

Adult Testis	Yes Leydig and germ cells	Yes Leydig and germ cells	


Bi potential XX gonad	Yes Germ and somatic cells	Yes Germ and somatic cells	Absent

Developing Ovary	Yes Germ cells	Yes Germ cells	Absent

Adult Ovary	Yes Somatic and germ cells	Yes Somatic and germ cells	Absent

### *ATRX *and *ATRY *mRNA distribution in the developing mammalian testis

Ubiquitous expression of *ATRX *in mice and *ATRY *in marsupials was detected in the indifferent XY gonad before sexual differentiation by whole mount *in situ *hybridization. Staining was weak and diffuse, and localized throughout the entire gonad and mesonephros.

**Figure 1:**
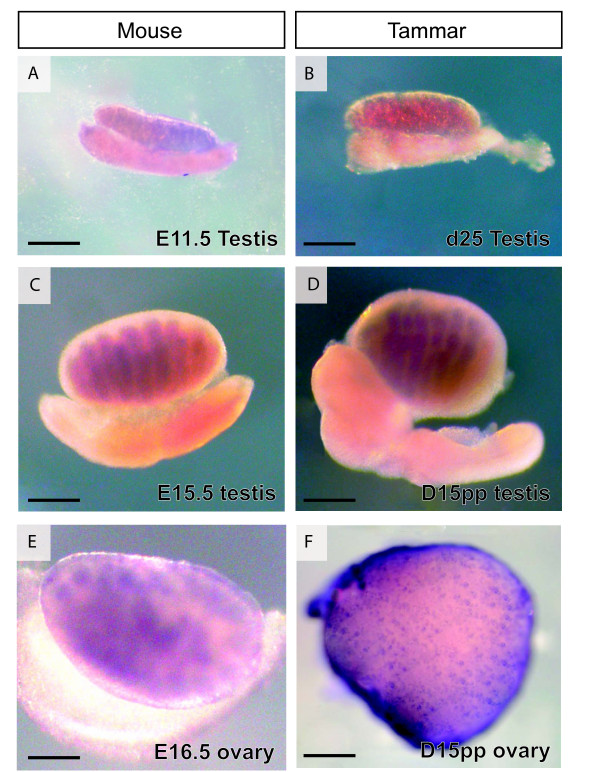
**Whole mount *in situ *hybridization of *ATRY *in the developing tammar testis (B, D) and *ATRX *in the developing mouse testis (A, C) and mouse and tammar ovary (E, F)**. Stages shown for mouse and tammar are at equivalent developmental time points. mRNA distribution is shown by dark blue/purple staining and tissues are bleached white. *ATRX *and *ATRY *mRNA transcripts were diffuse and dispersed throughout the indifferent gonad and to a lesser extent, the mesonephros (A,B). Once testicular differentiation began, *ATRX *in mouse and *ATRY *in tammar were confined to the developing testis cords and was absent in the regressing mesonephros (C, D). In the ovary, *ATRX *mRNA showed a punctate localization in the cortexin both mouse (E) and tammar ( F), indicative of germ cell staining. M - mesonephros, G - gonad, O - ovary, T - testis, scale bar = 500 μm.

During testicular differentiation, *ATRX *mRNA distribution in the mouse was identical to *ATRY *mRNA distribution in the tammar testes at all stages examined (Figure 1 A, B, C, D). We investigated the mouse from E11 through to E16.5 and equivalent development stages in the tammar wallaby testis from d24 of the 26.5 day gestation to day 40 post partum. The pattern of expression throughout these stages did not change from those shown in Figure 1. As development proceeded, *ATRX*/*Y *staining was lost in the mesonephros but persisted in the gonads. Intense staining was detected within the newly formed seminiferous cords (Figure 1C, D), suggesting localization of *ATRX*/*Y *transcripts in the germ cells and Sertoli cells of the testis throughout its development. Diffuse staining was also seen in the interstitial tissue suggesting expression outside the cords.

### ATRX and ATRY protein distribution in the developing mammalian testis

**Figure 2:**
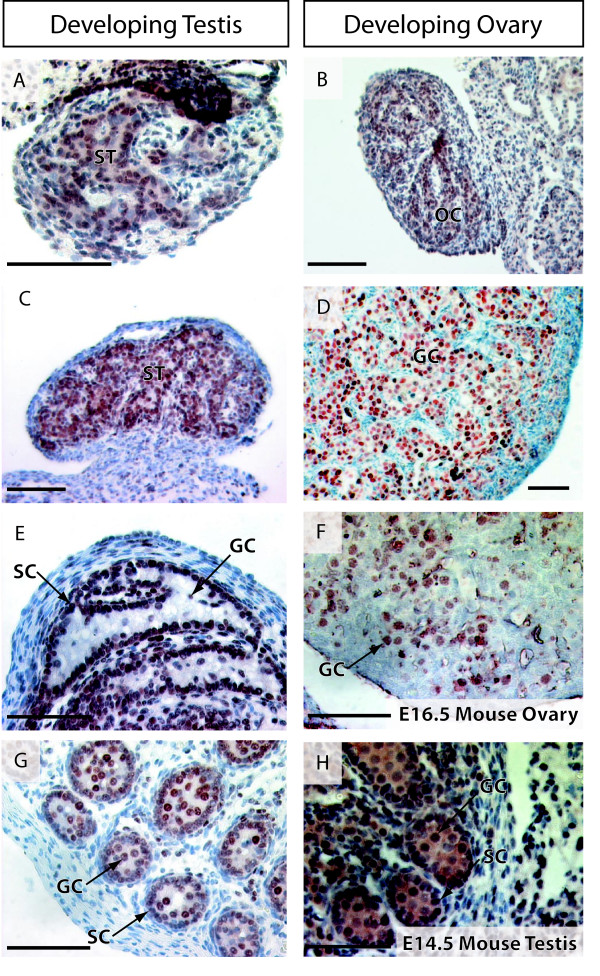
**Immunohistochemistry for ATRY in the tammar testis (A, C, E) and ATRX in the tammar ovary (B, D) and mouse ovary and testis (F, H)**. Antibody staining is shown by brown/red staining and tissues are counterstained blue with haematoxylin. In the developing tammar before gonadal sex determination, ATRX was confined to the primitive sex cords (A, B, developing seminiferous tubules in males or cortical cords in females) of both sexes. After testicular differentiation, ATRY protein was localized in the Sertoli cells and germ cells of the seminiferous tubules (C). This is clearly evident by day 8 post partum, where ATRY was clearly nuclear in both Sertoli and germ cells (E). By day 40 post partum the same pattern was seen as well as expression in a subset of interstitial cells that resemble Leydig cells (G). An identical protein localization was seen for ATRX in the mouse developing testis (H) with nuclear staining detected in the Sertoli cells, germ cells and some interstitial cells. After ovarian differentiation, ATRX protein was strongly nuclear and restricted to the germ cell lineage (D). An identical staining pattern was seen for ATRX in the mouse ovary (F). GC - germ cell, SC - Sertoli cell, ST - seminiferous tubule, CC - cortical cords, Scale bar = 500 μm.

ATRY protein was detected by the ATRX antibody in both the nucleus and the cytoplasm of the somatic and germ cells of the developing male tammar, at the time of gonadal sex determination (Figure 2A). By day 2 post partum, the cords are defined and ATRY had become nuclear and was restricted to the Sertoli and germ cells (Figure 2C). This expression pattern persisted throughout development with additional ATRY protein found in a subset of the interstitial cells (including cells with a Leydig like shape and location) in later stage gonads (Figure 2E,G). An identical staining pattern was observed for ATRX protein in the mouse testis at E14.5 (Figure 2H).

### ATRX and ATRY distribution in the adult tammar testis

**Figure 3:**
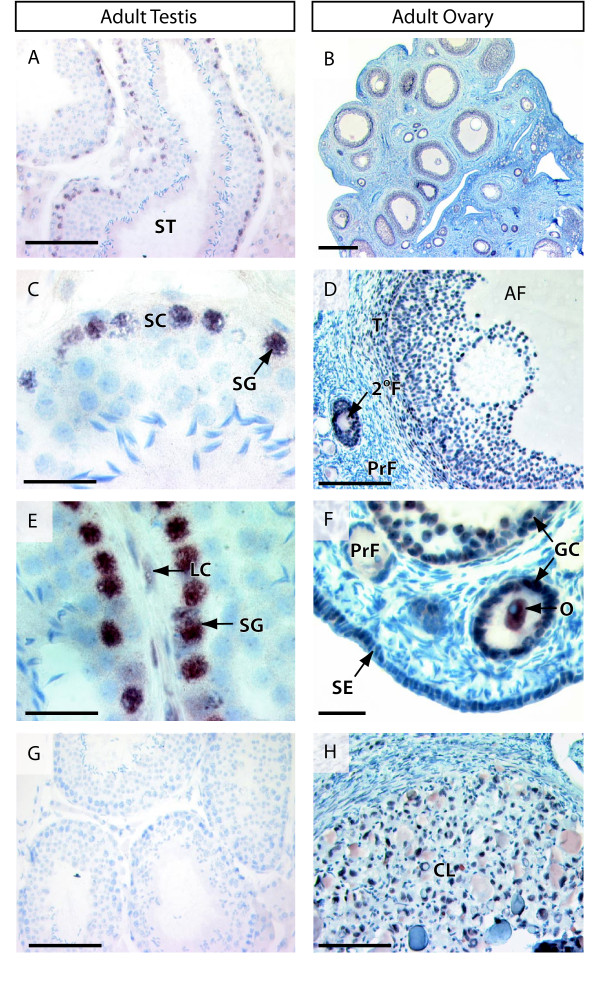
**Immunohistochemistry of combined ATRX and ATRY in the adult tammar wallaby testis, and for ATRX in the adult tammar ovary**. Antibody staining is shown by brown/red staining and tissues are counterstained blue with haematoxylin. In the adult testis, ATRX/Y was predominantly localized at the periphery of the seminiferous tubules (A). ATRX was nuclear in the primary spermatogonia but was absent from the Sertoli cells (C). Protein was also detected in the interstitial cells that resemble Leydig cells (E). In the adult ovary ATRX was seen in the granulosa cells of developing follicles (B) at all stages of growth, but not in primordial follicles (D, F). ATRX was also detected in the steroidogenic theca cells (D) of antral follicles, in the corpus luteum (H), in the ovarian surface epithelium (F) and in the oocytes themselves (F). There was no staining in the antibody negative control (G). SG - spermatogonia, GC - germ cell, SC - Sertoli cell, LC - Leydig cell, ST - seminiferous tubule, AF - antral follicle, T - theca, 2°F - secondary follicle, PrF - primordial follicle, GC - granulosa cell, O - oocyte, SE - ovarian surface epithelium, Scale bars; A,D,G,H = 200 μm, B = 500 μm, C, E, F = 50 μm.

Since ATRX and ATRY are co-expressed in the adult tammar testis [6], their differential protein localization was not able to be determined. ATRX/Y protein was present in the primary spermatogonia located at the base of the seminiferous tubules in the adult tammar testis (Figure 3A), but was lost from the mature Sertoli cell nuclei (Figure 3C). ATRX/Y protein was also detected in the interstitial cells (including cells with a Leydig like shape and location) and some - but not all - peritubular myoid cells (Figure 3E).

### *ATRX *mRNA distribution in the developing mammalian ovary

As in the testis, expression of ATRX and its protein localization in the developing ovary was identical between mice and marsupials. We investigated mouse stages from E11 through to E16.5 and equivalent stages in the tammar wallaby (from d24 of gestation to day 40 post partum). The pattern of expression in these stages did not change from those shown in Figure 1. A weak staining pattern was identified throughout the gonad at early developmental time points. After sexual differentiation, staining became more intense and punctate, indicative of protein within the germ cell nests (Figure 1 E, F). A low level of diffuse staining also persisted throughout the somatic cells of the ovary but this was at a much lower level than that observed in the germ cells (Figure 1 E, F). Thus the germ cells appear to be the major site of expression in the developing ovary. Again staining was observed in the mesonephros at early developmental time points (before gonadal sex determination) but was lost during gonadal development.

### ATRX protein distribution in the developing mammalian ovary

Staining in the indifferent ovary was both cytoplasmic and nuclear, and identical to the staining observed in the indifferent testis (Figure 2A), restricted largely to the germ cells and some somatic cells (Figure 2B). As the ovary developed, ATRX antibody strongly stained in the nucleus of XX germ cells and was completely absent from the somatic cell lineages (Figure 2D). An identical staining pattern was seen in the E16.5 mouse ovary, with ATRX protein exclusively localized to the nuclei of germ cells (Figure 2F).

### ATRX distribution in the adult ovary

ATRX protein persisted in the nuclei of developing oocytes but was also found at high levels in the nuclei of granulosa cells of growing follicles (Figure 3B, D). ATRX was not seen in the flattened granulosa cells of primordial follicles, but was abundant and nuclear in the cuboidal granulosa cells of follicles recruited to the growing pool (Figure 3F). ATRX was also detected in the luteal cells of the corpus luteum (Figure 3H) and the theca cells of large antral follicles (Figure 3D). In addition ATRX was nuclear and abundant in the ovarian surface epithelium (Figure 3F).

### *ATRX *mRNA distribution in the developing mammalian embryo

**Figure 4:**
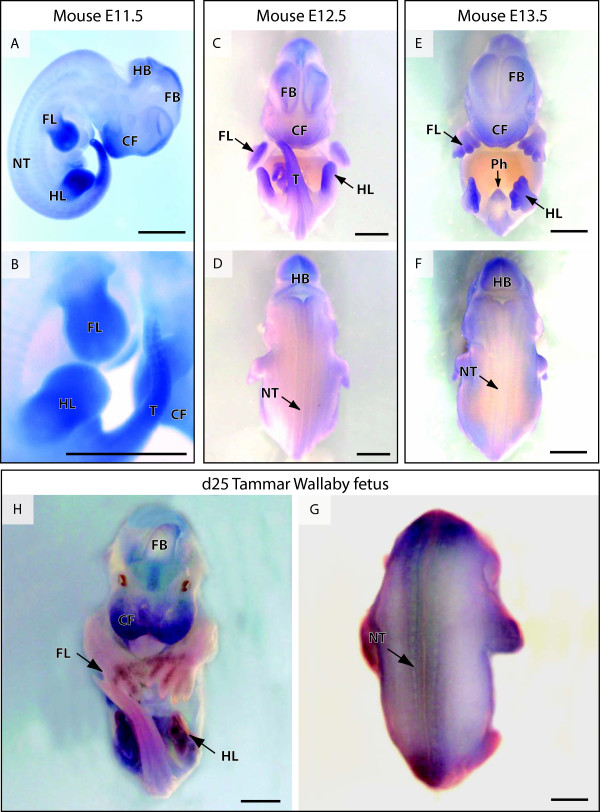
**Whole mount *in situ *hybridization of *ATRX *in the developing mouse and marsupial embryo**. mRNA distribution is shown by dark blue/purple staining, while tissues are bleached white. In the E11.5 mouse, mRNA was concentrated in the developing limbs, tail and craniofacial regions of the fetus. Staining was also evident in the brain, neural tube and dorsal root ganglia (A, B). mRNA persisted in each of these domains throughout mouse development and was distinct in the forming hand and foot plate, developing phallus, fore and hind brain, dorsal root ganglia, neural tube and craniofacial regions (C-F). Identical zones of mRNA expression were detected in the near-term tammar wallaby fetus (G,H). HL - hind limb, FL - fore limb, P- phallus, FB - fore brain, HB - hind brain, NT - neural tube, CF - craniofacial regions, T - tail. Scale bar = 2 mm.

**Figure 5:**
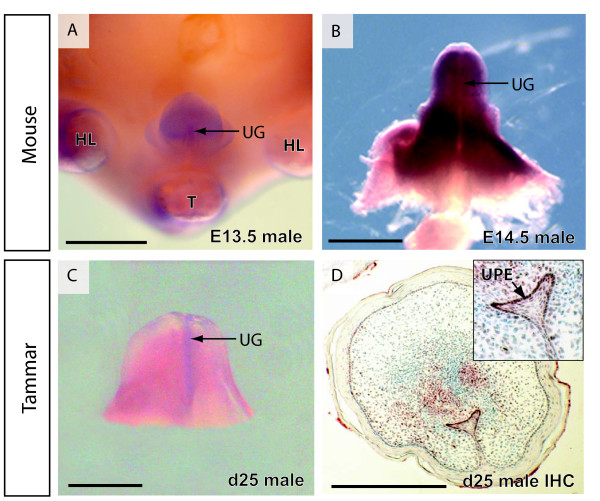
**Expression and protein distribution of ATRX in the phallus**. ATRX mRNA was seen throughout the developing genital tubercle in the male mouse (A, B). Hind limbs and tail were removed to allow clear observation of the phallus (A) or the entire phallus was removed (B,C). mRNA appeared to be more concentrated in the urethral groove on the ventral side of the phallus (A,B). Identical mRNA distribution was seen in the developing tammar phallus (C) again concentrated in the urethral groove. ATRX protein was distributed through cells of the tammar phallus mesenchyme but was concentrated in the urethral plate epithelium, lining the urethral groove (D) and enlarged insert (D). HL - hind limb, T - tail, UG - urethral groove, UPE - urethral plate epithelium. Scale bars; A,B = 1 mm and C,D = 500 μm.

*ATRX *mRNA distribution was identical and highly conserved in the mouse and tammar by whole mount *in situ *hybridization during early embryonic development. *ATRX *mRNA expression was pronounced in the developing fore- and hind-limbs, tail, craniofacial regions and developing brain. Staining in the craniofacial regions was consistently more intense in the anterior aspects where the nose and mouth are situated (Figure 4A, B). *ATRX *mRNA was also seen in the neural tube and dorsal root ganglia at all stages. ATRX expression persisted in all tissues described above through E13.5 in the mouse. In the tammar at day 25 of gestation (one day before birth) staining was prominent in the hind limb (equivalent in stage to an E13.5 mouse hind limb) but was fading in the forelimb, which is developmentally accelerated and equivalent to an E17.5 mouse forelimb (Figure 4C, D, E, F, G, H). ATRX expression was also prevalent in the developing phallus of both mice and marsupials (Figure 4E; Figure 5A, B, C, D). ATRX expression is concentrated at the ventral midline (urethral grove) and in the preputial swellings of the mouse phallus (Figure 5A, B). This staining pattern was present at each stage examined from E10.5 to E16.5 in mouse and day 22 to day 25 of gestation to in the tammar. Immunohistochemistry of the developing phallus showed ATRX protein was present in the surface epithelium, sporadically in the mesenchyme and intensely stained the epithelium of the forming male urethra up until at least day 8 post partum.

## Discussion

This is the first analysis of ATRX expression in the developing mammalian fetus, and demonstrates that the regional distribution of *ATRX *mRNA is highly conserved between eutherian and marsupial mammals. All sites of expression correspond with developmental deficiencies found in ATR-X patients [2,6] (Table 1). There was strong expression of *ATRX *in the developing brain, neural tube and dorsal root ganglia. These sites of expression are consistent with the psychomotor retardation, central nervous system and neural tube defects including spina bifida, scoliosis and kyphosis seen in ATR-X patients [2]. This suggests *ATRX *plays a highly conserved and fundamental role not only in the brain but in multiple parts of the developing central nervous system in all mammals. The expression in the dorsal root ganglia suggests *ATRX *may be essential for normal afferent nerve development and is consistent with hypotonia and some of the facial and limb muscle phenotypes observed in ATR-X patients.

*ATRX *expression was very strong in the developing fore- and hind-limbs of the mouse and tammar. This was especially pronounced in early limb bud stages in the hand and footplate, suggesting a function in limb patterning. Hand abnormalities are frequently observed in ATR-X patients including clinodactyly, brachydactyly, tapering of the fingers, drum stick phalanges, cutaneous syndactyly and overlapping of the digits [2]. Foot deformities are also observed including pes planus, talipes equinovarus and talipes calcaneovalgus [2]. Together with our expression data, these phenotypes suggest *ATRX *is essential in hand and footplate patterning in all mammals and that the phenotypes observed in ATR-X patients result from a combination of skeletal defects as well as hypotonia.

Many of the same patterning factors seen in the developing limb are also essential for phallus outgrowth and development [17]. *ATRX *fits into this class of factors as it was detected at high levels in the developing phallus. ATR-X patients frequently have penile abnormalities ranging from mild hypospadias and deficient prepuce to micropenis and severe hypospadias with ambiguous genitalia. The primary cause of these defects has been attributed to gonadal dysgenesis. However, the dynamic expression of ATRX in the developing phallus with mRNA and protein concentrated in the developing urethral plate (a signaling center for phallus patterning and urethral closure) suggests that at least a subset of these defects may be due to a direct role of ATRX in penile development and not simply a lack of androgen. Similarly, undescended testes, common in ATR-X patients, could also be the direct result of deficient genitofemoral nerve function or development [18], consistent with the expression detected in the dorsal root ganglia of developing embryos and may not necessarily be a secondary effect of androgen deficiency.

The role of ATRX and ATRY in gonadal formation appears to be identical between mice and tammar, indicating a highly conserved and fundamental role in gonadal development in mammals. We have previously shown that the marsupial specific Y-linked orthologue, *ATRY*, is exclusively expressed in the developing marsupial testis [6]. In mice, ATRX was present in the somatic and germ cells of the bipotential gonad of both XX and XY fetuses before sexual differentiation. Similarly, in marsupials, ATRX was present in the somatic and germ cells of the bipotential XX gonad and ATRY in the bipotential XY gonad prior to sexual differentiation. Despite their early expression, ATRX/Y does not appear to have a functional role in early gonadal formation since even the most severely affected ATR-X patients show signatures of normal early testis development. This is demonstrated by the absence of Müllerian ducts in ATR-X patients, confirming early testicular differentiation and the formation of functional Sertoli cells that secreted AMH [6].

After sexual differentiation and the initiation of testicular development, ATRX (in mice) and ATRY (in marsupials) became strongly nuclear and localized in the Sertoli and germ cells. This was consistent with *in situ *experiments showing mRNA localized in the forming seminiferous cords. The Sertoli cells are essential for maintaining testicular structure and function. Although ATRX may not be required for initial Sertoli cell development, the gonadal dysgenesis phenotypes seen in ATRX patients are consistent with deficient Sertoli cell maintenance. ATRX (in mice) and ATRY (in marsupials) also appears to have an important function in germ cell development as it is present in these cells in both mammals. However, testicular development and androgen secretion from the Leydig cells is still possible in germ cell deficient testes, suggesting that the lack of masculinization in ATR-X patients is not due to germ cell loss, but potentially a defect in the Leydig cells themselves. A recent study has shown the ATRX can enhance the expression of androgen-dependent genes through physical interaction with androgen receptor in a human Sertoli cell line [19]. In the absence of a definitive Leydig cell marker in the tammar wallaby testis, interstitial cells were classified on their location and morphology. Cells that morphologically resemble Leydig cells were ATRX positive (in mice) and ATRY positive (in marsupials) supporting the suggestion of a possible role in Leydig cell maintenance. However, we cannot exclude the possibility that the lack of androgen in ATR-X patients may be primarily caused by Sertoli cell-mediated gonadal failure, but the expression profile raises the possibility of a more direct role for ATRX (in mice) and ATRY (in marsupials) in androgen function.

Although the marsupial specific Y-linked orthologue, *ATRY*, is exclusively expressed in the developing marsupial testis [6], in the adult *ATRX *and *ATRY *mRNA are co-expressed and the ATRX antibody does not discriminate between the two orthologues. Interestingly, in the adult testis, ATRX/Y was lost from the Sertoli cell lineage suggesting that while it appears to be initially required to maintain Sertoli cells and early testicular viability, it is not required for mature Sertoli cell function. However, ATRX/Y remained localized in the germ cell derivatives and was primarily detected in the type A spermatogonia located at the basement membrane of the seminiferous tubules. Thus there may be a direct role of ATRX/Y in maintaining spermatogonial stem cells. Taken together, the complex expression of ATRX and ATRY in the developing and adult testis suggests it functions in multiple cell lineages in both the somatic and germ cell compartments.

Since it is a rare X-linked recessive condition, no female ATR-X patients have been identified to date, so a role for this gene in ovarian development has not been established. Our mRNA and protein localization analyses show that there is a highly conserved pattern of expression in the developing mammalian ovary. Initially, ATRX protein was cytoplasmic and nuclear, and was localized in the germ cells of the developing ovary (similar to the pattern seen in the indifferent XY gonad). A few days later, ATRX protein was pronounced and strictly nuclear in the germ cells that were congregated in nests. This was confirmed by whole mount *in situ *hybridization, showing punctate mRNA staining in the cortex of both the mouse and tammar ovaries, indicative of germ cell expression. There was no ATRX expression in the ovarian supporting cell lineage until puberty. ATRX was strongly nuclear in the granulosa cells of growing and atretic follicles. Interestingly, ATRX was absent in the flattened granulosa cells surrounding primordial follicles, but rapidly accumulated once the cells became cuboidal and the follicle was recruited to the growing pool. This suggests a potential role for ATRX in regulating granulosa cell growth during folliculogenesis. In addition to the supporting cells, ATRX was also present in the oocytes at all stages of follicular development. This is consistent with a continued role in germ cell maintenance as seen in the adult testis.

ATRX was also detected in the luteal cells of corpus luteum and in the theca cells of large antral follicles suggesting a possible function in steroidogenesis. In addition, ATRX was strong and nuclear in the ovarian surface epithelium where it may have role in proliferation and repair functions after ovulation. Interestingly, over 85% of ovarian cancers are derived from the ovarian surface epithelium [20] and ATRX has been shown to be upregulated in ovarian cancer cell lines [18].

The observed pattern of expression for ATRX and ATRY in the gonads of mice and marsupials was strikingly similar to that of DMRT1 in mice, humans and the tammar [16,21,22]. *DMRT1 *represents the most ancient and conserved gene in the sex determination pathway with orthologues involved in sexual development in flies and worms. *DMRT1 *is expressed at all the same stages of development as *ATRX *in mice and *ATRY *in marsupials and is distributed in the Sertoli and germ cells of the developing mammalian testis. In the adult, it is similarly lost from Sertoli cells but is maintained in the type A spermatogonia [16,22]. In the developing ovary, DMRT1 is present in the germ cells and then later seen in the granulosa cells and germ cells of the adult ovary [16]. The gonadal phenotype of ATR-X and DMRT1 patients is strikingly overlapping, with initial Sertoli cell development followed by gonadal dysgenesis [16,23-25]. Dmrt1 regulates germ cell proliferation in a cell autonomous and dosage dependent manner in mice, and its loss results in an increased incidence of teratomas [26,27]. In addition Dmrt1 is required to maintain Sertoli cell proliferation and viability [27]. Furthermore, haploinsufficiency of both *ATRX *and *DMRT1 *is compatible with normal ovarian development [5,21,25]. The strikingly similar protein distribution of ARTX and DMRT1 and the overlapping gonadal phenotypes (outlined in Table 1) between ATR-X and distal chromosome 9p deletion (including DMRT1) patients suggests a potential interaction between these two genes in the sexual development pathway. Furthermore, *ATRX*, like *DMRT1*, represents an ancient and ultra-conserved gene in the sex determination cascade. Thus, together *ATRX *and *DMRT1 *represent the most conserved somatic and germ cell factors in all animal species.

## Conclusion

All sites of ATRX/Y expression and protein distribution were consistent with phenotypes observed in ATR-X patients and suggest important roles for this gene in development of the CNS, limbs, phallus and gonads in all mammals. ATRX/Y expression and protein distribution in the gonad suggests it may regulate somatic and germ cell function and gametogenesis in both testicular and ovarian development. The conserved mammalian ATRX/Y expression pattern strikingly overlaps with that of DMRT1 suggesting an ancient and conserved interplay between these two genes in the development of the gonad. Finally, the similarity between the expression of *ATRX *and *ATRY *in mammals and the mutant *xnp-1 *phenotype in *C.elegans *indicates an ultra-conserved role for this gene in development and maintenance of the animal gonad [15,16,28].

## Methods

### Animals

Tammar wallabies *Macropus eugenii *from Kangaroo Island (South Australia) were maintained in open grassy yards in our breeding colony in Melbourne, Australia. Pouch young were removed from their mother's pouch for analysis. When the day of birth was uncertain, age was estimated using head length and published growth curves [29]. Fetal samples were obtained from pregnant females after removal of the pouch young (designated as day 0 of gestation) as previously described [30,31]. Fetal mice were obtained from pregnant females with the day of vaginal plug designated as day 0.5 of gestation [32] All sampling techniques and collection of tissues conformed to Australian National Health and Medical Research Council guidelines (2004) and were approved by The University of Melbourne Animal Experimentation & Ethics Committees.

### Tissues

Three replicates were performed for each tissue or fetus for the immunohistochemistry and *in situ *hybridization. All tissues were collected under RNase-free conditions fixed overnight in 4% paraformaldehyde, washed several times in 1 X PBS, and stored in 100% methanol at -20°C before paraffin embedding and sectioning at 8 μm for immunohistochemistry, or rehydrating in to PBS for *in situ *hybridization.

### mRNA *in situ *hybridization

Whole-mount *in situ *hybridisation was carried out using standard methods [33] Antisense and sense RNA probes were prepared separately from a region of ATRX spanning exons 10-12 that is highly conserved between ATRX and ATRY using the following primers; **Forward Primer: **5' CCTACTAAGCCTAAAGAGCAT 3'; **Reverse Primer: **5' TCAAGGAGGAGGATTATTTTA 3' and producing a product of 1113bp. Probes were labeled with digoxigenin-UTP, incorporated by either SP6 or T7 RNA polymerase. Whole mount *in situ *hybridisation was carried out in groups according to stage and sex, with varying proteinase-K digestion times ranging from 270 seconds for gonads before sexual differentiation to 15 minutes for E16.5 mouse ovaries and post partum tammar wallaby ovaries. Testes were digested twice to enable probe penetration of the thick tunica. Whole embryos and gonads were hybridised with the *ATRX/Y in situ *ribo-probe overnight at 65 C. The probe was detected with NBT/BCIP and photographs following dehydration and rehydration through a methanol series as described [32].

### Immunohistochemistry

Antigen retrieval was performed in boiling 0.05 M NaCit (pH 7.0) for 20 minutes. Sections were pre-treated with 3% hydrogen peroxide in methanol for 15 minutes. The ATRX primary antibody (rabbit anti-human, Santa Cruz biotechnology, ATRX-H-300) was applied to tammar adult testis tissue sections at 1:150 dilutions at 4°C overnight. Signal was amplified using the ABC/HRP kit (DAKO) visualized with AEC+ chromogen (Thermo Scientific), and counterstained briefly with haematoxylin.

## Authors' contributions

All authors participated in the design of the study. Tissue samples were collected by MBR and AP. AP and KH performed all the experiments. Results were analyzed by AP and KH who also drafted the manuscript. All authors read, modified and approved the final manuscript.
